# Has intraoperative neuromonitoring changed the surgery for unruptured middle cerebral artery aneurysms? A retrospective comparative study

**DOI:** 10.1007/s10143-023-02099-w

**Published:** 2023-08-03

**Authors:** Benjamin Skrap, Rina Di Bonaventura, Michele Di Domenico, Carmelo Lucio Sturiale, Anna Maria Auricchio, Rosario Maugeri, Giuseppe Roberto Giammalva, Domenico Gerardo Iacopino, Alessandro Olivi, Enrico Marchese, Alessio Albanese

**Affiliations:** 1 Department of Neurosurgery, Fondazione Policlinico Agostino Gemelli IRCCS, Largo Agostino Gemelli 8, 00168 Rome, Italy; 2 Institute of Neurosurgery, Catholic University of the Sacred Heart, Largo Francesco Vito, 1, 00168 Rome, Italy; 3 Neurosurgical Clinic, AOUP “Paolo Giaccone”, Department of Biomedicine Neurosciences and Advanced Diagnostics, School of medicine, University of Palermo, Palermo, Italy

**Keywords:** Aneurysm, MCA, MEPs, Neuromonitoring, SEPs, Temporary clipping

## Abstract

Intraoperative neurophysiological monitoring (IONM) represents one of the available technologies able to assess ischemia and aimed to improve surgical outcome reducing the treatment related morbidity in surgery for intracranial aneurysms. Many studies analyzing the impact of IONM are poised by the heterogeneity bias affecting the cohorts. We report our experience with IONM for surgery of unruptured middle cerebral artery (MCA) aneurysm in order to highlight its influence on functional and radiological outcome and surgical strategy. We retrospectively reviewed all MCA unruptured aneurysms treated between January 2013 and June 2021 by our institutional neurovascular team. Patients were divided into 2 groups according to the use of IONM. A total of 153 patients were included in the study, 52 operated on without IONM and 101 with IONM. The groups did not differ preoperatively regarding clinical status and aneurysm characteristics. Patients operated with IONM had better functional outcomes at discharge as well as at follow-up (*p*= 0.048, *p*=0.041) due to lower symptomatic ischemia and better radiological outcome due to lower rate of unexpected aneurysmal remnants (*p*= 0.0173). The introduction of IONM changed the use of temporary clipping (TeC), increasing its average duration (*p*= 0.01) improving the safety of dissecting and clipping the aneurysm. IONM in surgery for unruptured MCA aneurysm could improve the efficacy and safety of clipping strategy in the way it showed a role in changing the use of TeC and was associated to the reduction of unexpected aneurysmal remnants’ rate and improvement in both short- and long-term patient’s outcome.

## Introduction

Middle cerebral artery (MCA) aneurysms represent around 20% of all intracranial aneurysms [1]. They generally arise at the bifurcation, thus surgical clipping still represents the gold standard of treatment since several studies claimed its superiority over endovascular in obtaining complete occlusion with distal flow preservation. MCA aneurysms’ clipping it’s not merely an exclusion of the sac but rather the reconstruction of the bifurcation [2]. Surgery compared to endovascular has lower risk of thromboembolism of the perforators and related hemorrhagic infarction [2–9] and long-term recurrence/retreatment [10].

Due to relatively low risk of rupture [11] the treatment for intact aneurysms should ideally implicate a very low risk of complications. Ischemia, the most feared by neurosurgeons [12, 13], is reported in about 7% of cases [14].

Therefore, intraoperative techniques that allow to ascertain both the aneurysm complete occlusion and the preservation of parent arteries are essential, as they allow to adjust the surgical strategy in order to avoid ischemic complications [15–17].

Various techniques have been developed for this purpose, to assist surgery and integrate visual inspection in place of intraoperative Digital Subtraction Angiography (DSA) which is time consuming and requires a hybrid theatre: Microdoppler (MCD) and Indocyanine Green Video-Angiography (ICG-VA) play the major role [18, 19]. These direct flow-preservation techniques are able to point out a reduction in flow but they cannot evaluate its impact on brain [14–17, 20–22]. On the other hand Intraoperative Neurophysiological Monitoring (IONM) is able to indirectly detect and prevent ischemic injury during surgery [18, 23]. Its accuracy has been validated [17] but its clinical impact and relevance for aneurysms is still debated as the majority of available studies are retrospective and heterogenous [21, 24].

## Objectives

We reviewed the use of IONM in our institutional series of unruptured MCA aneurysms to study its influence on clinical and radiological outcome. Furthermore, we aimed to point out how IONM has changed the clipping strategy.

## Materials and methods

We retrospectively reviewed our database of surgically treated unruptured MCA aneurysm between January 2013 and June 2021. IONM was introduced in our institution in 2013 but it was routinely used for vascular neurosurgery since 2017 due to the availability of a dedicated neuromonitoring team and suite. Therefore only a part of clipping procedures performed between 2013 and 2017 was IONM-assisted. All cases were operated by the same full trained neurovascular team. For all patients we reviewed clinical, surgical and neuromonitoring reports. We divided patients in two groups: Group 1=non IONM-assisted surgery and Group 2= IONM-assisted surgery.

All patients gave their informed consent to use their anonymized data. Ethics committee approval was not requested in our Institution due to the retrospective and anonymous nature of the study. The neurological status pre-, postoperatively and at follow-up was classified according to the modified Rankin Scale (mRS). Any new neurological deficits or seizures occurring during in-hospital stay and follow-up was recorded.

Each patient had at least one Computed Tomography Angiogram (CT) pre-, post-operatively (<72h after surgery) and at follow-up. We analyzed the aneurysm characteristics (dome and neck size, their ratio, presence of blebs), the surgical outcome based on complete/uncomplete aneurysm occlusion, radiological and clinical ischemia and related neurological complications (deficits and seizures). The sac remnant knowingly left during surgery (described in the surgical report) was classified as “expected”. Any new brain hypodensity in the MCA territory at postoperative CT was reported as radiological ischemic sign.

### Surgery

Since 2014 we progressively adopted the minipterional approach for all elective MCA aneurysms due to better patient-oriented outcomes and lower complications rate [25]. We adopt an exclusively “retractorless” technique [26]. The sylvian fissure dissection is performed with a mixed sharp-blunt technique and it measures 1.5–2 cm on the horizontal part of the fissure. Once completed the aneurysm dissection, we study MCD. After clipping, we follow a multimodal flow preservation-aimed protocol based consecutively on visual inspection, MCD and ICG-VA in a step-by-step procedure.

For this study we collected data regarding the surgical time, the reconstruction technique classified in simple (1 or 2 stacked clips orthogonal to the neck), or complex (3 or more clips, tandem or composite clipping or wrapping), duration and repetition of temporary clipping (TeC) to obtain proximal control and final clip repositioning rate and its reason (alerts from visual inspection, MCD, ICG-VA or IONM).

### IONM

IONM proceeds from skin incision till at least 30 min after final clipping. Evoked potentials at the beginning and at the end of surgery, their changes during TeC and after final clipping and their influence on surgical strategy are described in detail in neuromonitoring reports. All procedures were assisted by a Medtronic NIM-Eclipse neuromonitoring suite. The IONM technician, with a specific neurosurgical preparation and deep knowledge of neurosurgical procedures was present during the whole surgery. Our standard IONM protocol for unruptured MCA aneurysms includes motor-evoked potential (MEPs) and somatosensory-evoked potentials (SSEPs) of the contralateral side and EEG.

MEPs were elicited via transcranial stimulation and recorded with needle electrodes into upper and lower extremity muscles (Bicipitis brachialis, Extensor carpi ulnaris, Abductor pollicis brevi, Quadriceps femori, Tibialis anterior). Short trains of 5–7 square-wave stimuli (multipulse technique, 100–400 Volts, duration of 0.5 ms, and interstimulus interval of 2–4 ms) were delivered through cork-screw electrodes at C1 or C2 scalp sites (used as anode to elicit right or left limbs MEPs respectively). For MEPs we used closed-to-motor-threshold stimulation and the most-focal stimulating electrode montage to limit the activation of subcortical structures that could lead to false negative results and to minimize patient movement allowing MEPs acquisition without interrupting the surgery [24, 27]. SEPs were elicited by stimulation of the median nerve at the wrist and the posterior tibial nerve at the ankle (intensity 15–40 mA, duration 0.5 ms, repetition rate of 4.1 Hz). Recordings were performed via cork-screw-like electrodes inserted in the scalp at CZ′-FZ (legs) and C3′/C4′-FZ (arms) (according to 10–20 International EEG system). EEG is recorded, during surgery, from three bipolar channels: C3′–Fz, Cz′-Fz, and C4′–Fz. We benefit from the use of EEG for evaluation of deepness of anesthesia and reliability of intraoperative neuromonitoring.

Wave disappearence or >50% amplitude reduction was considered significant changes for MEPs, whereas disappearance, >50% reduction in amplitude, and/or 10% increased latency were considered significant changes for SEPs. The surgeon was always warned even when minor modifications occurred.

### Anesthesiological protocol for IONM

Inhalational agents and muscle relaxants are confounders for MEPs monitoring as they have deleterious effects on the amplitude of the waveform signal. SSEPs are also moderately affected. Totally intravenous anesthesia (TIVA) with no intraoperative muscle relaxants following intubation is the preferred technique for IONM assisted surgeries. Indeed TIVA regimes help to maintain the level of anesthesia during the critical monitoring periods in order to avoid confounding the interpretation of changes. Target Controlled Infusions (TCI) are a type of TIVA consisting of computer driven infusions to achieve a preset plasma concentration. Multi-compartment pharmacokinetic models are used to calculate infusion rate required to achieve the target concentration. Our TIVA/TCI protocol during IONM assisted surgeries is based on the use of Propofol (3.5mcg/mL) and Remifentanil (2–5 ng/mL).

### Statistical analysis

Statistical analysis was performed using Excel v. 16.53 for Mac (Microsoft, Redmond, WA, USA) and R statistical software’s (R version 4.1.1 (2021-08-10) “Kick Things”). The normality test was Shapiro-Wilk’s; continuous variables were compared with t student test while categorical variables were compared using the two-sided Fisher’s exact test. A *p*-value <0.05 was set as significant.

## Results

Between January 2013 and June 2021 181 patient underwent surgery for unruptured MCA aneurysms. We excluded multiple aneurysms clipped during the same surgery and recurrent aneurysm thus we included 153 patients for the final analysis. Group 1 patients (*n*=52) were operated without IONM assistance whereas group 2 (*n*=101) surgeries were IONM-assisted.

### Patients and aneurysms’ characteristics

Our series overall included 153 patients with mean age of 60.2±10.9 harboring unruptured MCA aneurysms. Among them 116 were women and 37 men. There were no statistical differences among the groups according to demographics (Table 1). The aneurysm discovery was incidental in 146 patients who were in good neurological status whereas 7 had had a previous subarachnoid hemorrage from the rupture of another aneurysm. Among these 7 patients, 5 had preoperative mRS 2 and 1 mRS 3. There was no statistical difference in the preoperative neurological status between the groups (*p*= 0.18) (Table 1).

**Table 1:** Patient characteristics: demographics and preoperative neurological status

		Study population *n*=153	Group 1 (non IONM) *n*=52	Group 2 (IONM) *n*=101	*p*-value
Sex	F	116 (75.8%)	39 (75%)	77 (76.2%)	
	M	37 (24.2%)	13 (25%)	24 (23.8%)	0.86
Age (mean±SD)		60.2±10.9	60.3±10.9	59.9±11.1	0.85
Preoperative mRS	0–1	147 (96.1%)	48 (92.3%)	99 (98%)	
	2–3	6 (3.9%)	4 (7.7%)	2 (2%)	0.18

*IONM* intraoperative neurophysiological neuromonitoring, *F* women, *M* men, *mRS* modified Rankin Scale

The two groups were similar according to the most relevant angioarchictectural characteristics of the aneurysms which are listed in Table 2.

**Table 2:** Aneurysm’s characteristics

		Study population *n*=153	Group 1 (non IONM) *n*=52	Group 2 (IONM) *n*=101	*p*-value
Side	L	63 (51.2%)	20 (47.6%)	43 (42.6%)	
	R	90 (48.78%)	32 (52.4%)	58 (57.4%)	0.62
Topography	M1	15 (9.8%)	6 (12.5%)	9 (9.3%)	
	Bifurcation	125 (81.7%)	41 (85.4%)	84 (86.6%)	
	Distal	5 (8.5%)	1 (2.1%)	4 (4.1%)	0.92
Aneurysm sac size mm (median; range)		5.4; 1–43	5.4; 2–43	5.1; 1–25	0.86
Aneurysm neck size mm (median; range)		3.4; 1–11	3.4; 2–7.5	3; 1–25	0.72
Aspect ratio (median; range)		1.6; 0.2–8.6	1.6; 0.7–8.6	1.5; 0.2–4.2	0.93
Blebs		70 (45.7%)	21 (40.4%)	49 (48.5%)	0.51
Perforators		22 (14.4%)	6 (11.5%)	16 (15.8%)	0.58

*IONM* intraoperative neurophysiological monitoring, *L* left, *R* right

### Surgery

Table 3 depicts the main aspects of surgery. The adoption of IONM did not influence the lenght of the surgical procedure (*p*=0.62). The number of patients who underwent a minipterional approach was significantly higher in group 2 as it collected the majority of the most recent cases. In group 2 (IONM-assisted) there was a higher number of aneurysms reconstructed with multiple clips (*p*= 0.039). The use of a single or multiple TeC was not in general statistically different between the groups but the permitted occlusion time was significantly longer for group 2 (*p*= 0.0005 and 0.01). One patient in group 1 whose aneurysm was clipped during TeC had a new postoperative deficit, whereas none of group 2 patients whose surgery was assisted by TeC had new postoperative deficit.

**Table 3:** Surgery

		Study population *n*=153	Group 1 (non IONM) *n*=52	Group 2 (IONM) *n*=101	*p*-value
Surgical time in minutes (median; range)		210; 120–420	210; 120–360	210; 120–420	0.62
Surgical approach	Minipterional	89 (58%)	8 (15%)	82 (81%)	
	Pterional	64 (42%)	44 (85%)	19 (19%)	<0.00001
Clipping technique	Simple	114 (74.5%)	44 (84.6%)	70 (69.3%)	
	Complex	39 (25.5%)	8 (15.4%)	31 (30.7%)	0.039
TeC use		33 (21.5%)	11 (21.2%)	22 (21.8%)	0.92
Multiple TeC		5 (15%)	1 (9%)	4 (18%)	0.49
Single TeC time (mean±SD ) min		4.1±2.5	2.5±1.8	5±2.8	0.0005
Total TeC time (mean±SD) min		8.2±5.2	3.2±1.8	8.2±5.9	0.01

*TeC* temporary clipping

### IONM

IONM was successfully performed in all group 2 patients. Table 4 describes intraoperative neuromonitoring changes and surgical actions in response to IONM alerts.

**Table 4:** IONM alerts during surgery and consequent surgical actions

Event		Surgical step	Surgical action
MEPs decrease	9 (8.9%)	2 during Tec	TeC interruption
		7 after final clipping	Clip repositioning
SSEPs	2 (2%)	2 during TeC	TeC interruption

*IONM* intraoperative neurophysiological monitoring, *MEPs* motor-evoked potentials, *SSEPs* somato-sensory-evoked potentials, *TeC* temporary clipping

### Temporary clipping

TeC was interrupted in 4 cases (12.5%) because of IONM alert: in two patients, MEPs decrease occurred after 6 and 10 min respectively; in two patients, MEPs and SEPs decrease occurred after 5 and 9 min respectively. In all these cases, evoked potentials came back to baseline after removal of the clip. None of these patients experienced a new postoperative deficit.

### Definitive clipping

Aneurysm’ clips were repositioned in 25 patients (24.7%). In 12 (48%), the clip was repositioned after surgeon’s visual inspection, in 7 (28%) after IONM changes (MEPs disappearance in 4, decrease in 3), in 4 (16%) after MCD’s evidence of parent vessel occlusion/stenosis, and in 2 (8%) after ICG-VA evidence.

In 5 out of 7 cases, the repositioning led to return to baseline of MEPs. None had a new postoperative deficit but one developed CT hypodensity and seizures.

All group 2, patients had continuous EEG monitoring during surgery. Six patients had perioperative seizures. Among them, just in one case a generalized seizure was observed during surgery. No case required IONM interruption.

### Aneurysm occlusion

The overall rate of complete occlusions was 85% (Table 5), similar in the two groups. In 16 cases, a residual of neck was left on purpose to avoid a flow obstruction (“expected” remnants), whereas in 7 cases (5 in group 1 and 2 in group 2) we observed an unexpected remnant on post operative angio CT scan. The rate between unexpected and expected remnants was significantly lower in the IONM group (*p*-value 0.0173) (Table 5).

**Table 5:** Aneurysm occlusion and postoperative ischemic changes

	Study population *n*=153	Group 1 (non IONM) *n*=52	Group 2 (IONM) *n*=101	*p*-value
Complete occlusion	129 (85%)	44 (84.6%)	86 (85.2%)	1
Unexpected/expected remnant	7/16	5/3	2/13	0.0173
Radiological ischemia	42 (27.4%)	14 (27%)	29 (28%)	0.52
Symptomatic ischemia	12 (7.8%)	8 (15.4%)	4 (3.9%)	0.01324

### Postoperative clinical and radiological outcome

Ischemic signs on postoperative CT were revealed in 42 patients (27.4%) (Table 5). Among them, just 12 (7.8%) had related clinical symptoms. Symptomatic ischemia was significantly less frequent in group 2 (*p*-value 0.01324). In this group 3, patients were symptomatic for speech disturbances and 1 for seizures; whereas in group 1, 5 patients had paresis (in two cases associated to aphasia), 2 had aphasia, and 1 seizures. Nine patients (5.8%) were discharged with new neurological deficits: 6 in group 1 (11.5%) and 3 in group 2 (2.9%) (*p*-value 0.033).

At dismissal, 5 patients from group 1 had hemiparesis, 1 aphasia. All 3 patients in group 2 with post operative speech disturbances were stable at dismissal. In group 2, no patients developed neurological deficits that could be prevented by IONM (Table 6).

**Table 6:** Clinical outcome at discharge and follow-up

		Study population *n*=153	Group 1 (non IONM) *n*=52	Group 2 (IONM) *n*=101	*p*-value
At discharge	New neurological deficits	9 (5.8%)	6 (11.5%)	3 (2.9%)	0.033
	Seizures	11 (6.7%)	5 (9.6%)	6 (5.9%)	0.61
	mRS 0–1	137 (89.5%)	43 (82.7%)	94 (93.1%)	0.048
At last follow-up	mRS 0–1	143 (93.5)	46 (88.4)	97 (96.1)	0.041

*mRS* modified Rankin Scale

The number of patients who experienced a good outcome was significantly higher in group 2. The occurrence of seizures was higher after pterional approach compared to minipterional (*p*= 0.027).

Globally 84% of patients was discharged with an excellent outcome (mRS 0–1): 82.7% in group 1 and 93.1% in group 2 (*p*= 0.048).

### Follow-up

At last follow-up (average 28 months), 88.4% of group 1 and 96.1% of group 2 had mRS grade 0–1 (*p*-value 0.041). Among patients discharged with a new deficit, 5 had complete recovery (mRS 0), 2 limited recovery (mRS 1–2), and 2 no recovery (mRS 4). In all but one case, the remnant aneurysm was stable and no patient experienced new related hemorrhages.

## Discussion

The IONM appears as one of the two pillars of the evolution of microvascular neurosurgery with miniaturization of cranial approaches and cerebrovascular dissection. Ischemic brain injury after intracranial aneurysm clipping is the most feared complication, occurs in up to 7% of cases and represents still the major challenge for the surgeon. IONM integrates assisting techniques of direct evaluation of flow (MCD and ICG-VA) during surgery because the ischemic risk depends not only on extent of flow decrease but also on its duration and brain demand.

The pioneering use of SSEPs in vascular neurosurgery was reported by Schramm in 1990 [16]. Szelenyi [23] and Neuloh [18] reported the advantage of combined use of SSEPs and MEPs in predicting new neurological deficits. In the last 2 decades, literature about the use of IONM in vascular neurosurgery was focused mainly on its predicting value. Byoun highlighted the usefulness of IONM during TeC, as it promptly detects an incipient cerebral ischemia preventing irreversible damage [20]. However, systematic reviews and metanalysis were unable to definitely support or refute the diagnostic value of IONM because of modest quality of data and the non-homogeneity among them [21, 25]. The main bias of these studies consisted in the heterogeneity of cohort including all location [28] or ruptured and unruptured aneurysms [14]. Yue et al. [14] conducted a prospective study with ruptured and unruptured MCA aneurysm showing better long-term outcome with IONM. Nasi et al. in his metanalysis evidenced a positive impact on outcome with IONM especially in MCA cohort [21]. Surgical experience in MCA aneurysms has been teaching us that IONM is able to guide the clipping strategy and to improve patient functional outcome. In our series of unruptured MCA aneurysms, IONM-assisted surgical cases had a significantly lower incidence of new deficits and better outcome both at discharge and follow-up and a statistically significant reduction of symptomatic brain ischemia [13]. The only new deficit in IONM group was aphasia that is unmonitorable during asleep procedures. Nevertheless some authors have proposed awake clipping strategy [29] but concerns have been expressed [30, 31] and its indication is controversial.

### IONM and surgical strategy

TeC is fundamental to reduce the risk of rupture during dissection, especially for complex aneurysms, but it can induces pre-ischemic/ischemic alterations with risk of subsequent deficits [28, 32, 33]. The maximum safe time of parent vessel’s temporary occlusion depends on patient’s reserve, vascular territory and anesthesia protocol [28, 33, 34]. In this context, IONM is the ideal technique to alert the surgeon of an impending ischemia with a high specificity [33].

In our series, the TeC rate was the same in the groups but the partial and total time of temporary occlusion increased in the monitored group because it is based on a real and not presumed ischemic alert provided by the IONM. The maximum safe TeC time can be highlighted by IONM: this allows the surgeon to proceed with more confidence and calm. On the contrary without IONM the surgeon miss an objective measure of TeC tolerability [18].

In group 2, definitive clip was repositioned in 25 patients, in 7 after IONM changes that occurred after our visual/micro-doppler and ICG-VA inspection protocol revealing false negatives of these techniques that would have probably led to additional morbidity. The limits of these techniques are the qualitative, at best semiquantitative, measure of flow, small vessels as perforators [18, 35] and collateral circulation/reservoir [18]. As already reported, vessels that are behind the aneurysms cannot be visualized by ICG-VA thus unable to diagnose their incidental closure [19, 36]. On the contrary IONM gives a real time continuous measure of function related blood flow. In our series IONM is the second cause of definitive clip repositioning after visual inspection. According to Neuloh et al. [18] IONM led to a change in strategy in 20% of cases and IONM+MCD led to a change of strategy in 33% of cases which is in line with our results. Della Puppa et al. [13] reported a rate of clip repositioning of 40.6%: because of MEPs in 9.3% of cases, due to MCD in 22.91% and ICG-VA in 8.3%. Both these studies share the heterogeneity bias including different locations aneurysms and both ruptured and unruptured.

### Occlusion rate and vessel reconstruction

Our occlusion rate is similar to that reported in literature [13]. In the monitored group the rate of complete obliteration is marginally higher and there’s a statistically significant reduction of unexpected remnant. Another relevant finding in our series was the evidence of the increased number of multiple clips reconstructions in IONM group. This was probably due to the combination of two factors namely the non-significantly higher number of complex aneurysm in group 2 and our attempt to obtain a more complete aneurysm exclusion supported by the continuous absence of IONM alerts.

These data suggest that IONM assisted surgery allows to increase completeness and safeness of the vascular reconstruction.

## Limitations

The main limit of this study lies in its retrospective nature thus the non randomization of the patients.

The majority of patients were evaluated with CTA: DSA, still the gold standard especially for small remnants, was available just for few patients. Our radiological criteria for ischemia were based solely on basal CT and they probably miss specificity.

## Conclusions

In our institutional series, the introduction of IONM in surgical clipping for unruptured MCA aneurysm has led to an improvement of both short- and long-term patient’s outcome, has changed the use of TeC, and has had a role in reducing the rate of unexpected aneurysmal remnants.

Neuromonitoring guides cerebrovascular surgery as well as mapping guide brain tumor surgery with the aim of maximizing aneurysm exclusion and minimizing the post operative neurological deficits.

## Data Availability

Not applicable.
