# Correction to: Has intraoperative neuromonitoring changed the surgery for unruptured middle cerebral artery aneurysms? A retrospective comparative study

**DOI:** 10.1007/s10143-023-02117-x

**Published:** 2023-08-24

**Authors:** Benjamin Skrap, Rina Di Bonaventura, Michele Di Domenico, Carmelo Lucio Sturiale, Anna Maria Auricchio, Rosario Maugeri, Giuseppe Roberto Giammalva, Domenico Gerardo Iacopino, Alessandro Olivi, Enrico Marchese, Alessio Albanese

**Affiliations:** 1 Department of Neurosurgery, Fondazione Policlinico Agostino Gemelli IRCCS, Largo Agostino Gemelli 8, 00168 Rome, Italy; 2 Institute of Neurosurgery, Catholic University of the Sacred Heart, Largo Francesco Vito, 1, 00168 Rome, Italy; 3 Neurosurgical Clinic, AOUP “Paolo Giaccone”, Department of Biomedicine Neurosciences and Advanced Diagnostics, School of medicine, University of Palermo, Palermo, Italy

**Correction to: Neurosurgical Review**

10.1007/s10143-023-02099-w

The authors regret that Dr. Michele Di Domenico was inadvertently omitted from the author list of the original article.

The original article has been corrected.

